# Cardiovascular Health Trends in Electronic Health Record Data (2012–2015): A Cross-Sectional Analysis of The Guideline Advantage™

**DOI:** 10.5334/egems.268

**Published:** 2019-07-18

**Authors:** Joyce E. Rudy, Yosef Khan, Julie K. Bower, Sejal Patel, Randi E. Foraker

**Affiliations:** 1 The Ohio State University, US; 2 The American Heart Association, US

**Keywords:** public health, epidemiology, risk factors, cardiovascular diseases, electronic health records

## Abstract

**Background:**

Electronic health record (EHR) data can measure cardiovascular health (CVH) of patient populations, but have limited generalizability when derived from one health care system.

**Objective:**

We used The Guideline Advantage™ (TGA) data repository, comprising EHR data of patients from 8 diverse health care systems, to describe CVH of adult patients and progress towards the American Heart Association’s (AHA’s) 2020 Impact Goals.

**Methods:**

Our analysis included 203,488 patients with 677,733 encounters recorded in TGA from 2012 to 2015. Five measures from EHRs [cigarette smoking status, body mass index (BMI), blood pressure (BP), cholesterol, and diabetes mellitus (DM)] were categorized as poor/intermediate/ideal according to AHA’s Life’s Simple 7 algorithm. We presented distributions and trends of CVH for each metric over time, first using all available data, and then in a subsample (n = 1,890) of patients with complete data on all metrics.

**Results:**

Among all patients, the greatest stride towards ideal CVH attainment from 2012 to 2015 was for cigarette smoking (50.6 percent to 65 percent), followed by DM (17.3 percent to 20.7 percent) and BP (21.1 percent to 23.2 percent). Overall, prevalence of ideal CVH did not increase for any metric in the subsample. Males slightly improved in ideal CVH for BMI and cholesterol; meanwhile, females saw no improvement in ideal CVH for any metric. As ideal CVH for BP and cholesterol increased slightly among white patients, ideal CVH for BP, cholesterol, BMI, and DM worsened for non-whites.

**Conclusion:**

Despite improvements in some CVH metrics in the outpatient setting, more tangible progress is needed to meet AHA’s 2020 Impact Goals.

## Introduction

Heart disease is the leading cause of mortality for men and women in the United States (US), accounting for 610,000 annual deaths [1]. It ranks second among the leading causes of years of life lost with an estimated 7,529,750 life-years lost in 2015 in the US [2]. Cardiovascular disease (CVD)-associated health care results in $207 billion US dollars spent annually on treatments and lost productivity [1]. A high prevalence of key CVD risk factors coupled with a low prevalence of ideal cardiovascular health (CVH) among US adults contribute to this epidemic [1,3]. It is critical to monitor and manage risk factors for CVD to reduce heart disease burden and to improve population CVH. Thus, health care providers and patients each play an important role in delivery of and adherence to guideline-based care for ultimately attaining ideal CVH in the population.

Electronic health record (EHR) data are increasingly used to assemble patient cohorts and conduct epidemiologic research on CVD risk factors [4]. While designed to aid in patient care and billing, EHR data provide demographic and CVD risk factor data which can characterize CVH in a variety of patient populations for epidemiological research [5,6,7,8]. From a public health perspective, measuring and monitoring CVD risk factors using EHR data can enhance surveillance efforts to identify patients at risk, plan initiatives to reduce cost and improve health outcomes, and guide policies and treatment guidelines to minimize heart disease burden [7,9,10]. Benefits of using EHR data include reduced study cost and time, and provision of data that can be used cross-sectionally, longitudinally, or for comparative effectiveness studies [4].

Previous population-based studies measuring CVH in the US have limitations in their design and scope. Cross-sectional national surveys have inherent issues with self-reported data and/or establishing temporality between risk factors and disease, and often do not reflect data collected in real-time [3,11]. EHR data can provide reliable, up-to-date information on CVD risk factors, yet results from a single health system may have limited generalizability across patient populations [12]. When EHR data are combined across health systems and time periods, external validity of the data can be enhanced and trends in CVH can begin to be elucidated [4]. To this end, the American Heart Association (AHA), the American Diabetes Association (ADA), and the American Cancer Society launched The Guideline Advantage™ (TGA), an outpatient quality improvement program [13]. Along with this, a data repository was created that represents the integration of data from EHRs across the US and is designed to measure adherence to clinical guidelines for prevention and management of chronic diseases in outpatient care settings [13]. For this study, we used TGA data to evaluate trends over time in CVH, and progress towards AHA’s 2020 Impact Goals “to improve the cardiovascular health of all Americans by 20 percent while reducing deaths from cardiovascular diseases and stoke by 20 percent [14].” We hypothesized that cigarette smoking, blood pressure, and cholesterol would improve, and obesity and diabetes mellitus would worsen over time [15].

## Methods

### Design Overview

We conducted a cross-sectional analysis of TGA data to describe the CVH of US adults from 2012 to 2015. TGA was initiated in 2011 to determine how consistent promotion of evidence-based practice guidelines aid in the prevention and management of chronic diseases [16]. Its repository is comprised of EHR data aggregated at a centralized data warehouse, where it is then advanced from clinical and health care purposes to actionable information for population health improvement [13]. TGA data have been collected retrospectively from 2010 and prospectively since 2011. TGA database contains demographic and health measures from EHRs of diverse patients representing all four US census regions and various health insurance types, including Medicare, Medicaid, and private insurers, as well as uninsured patients. An “open cohort” was assembled from patients of 8 geographically diverse health care systems that have elected to join the initiative over time. Patients were included in the cohort if they had at least one clinical encounter in the participating health care system(s) since TGA data collection began. Since health care systems have joined TGA over time, the cohort and participating health care systems has grown in number accordingly. Patients can exit the cohort by moving their care to a different health care system not participating in TGA, refusal to continue to receive medical care, or death. All clinics within a health care system were eligible for providing EHR data, but information on the number of clinics and names of participating health care systems were not disclosed. While health care systems were not required to submit certain data elements, participating health care systems were encouraged to submit all EHR data, especially those congruent with TGA’s ideal measure set, a list of measures needed for the evaluation and improvement of outpatient treatment.

### Measures and Data Sources

CVH data was provided from 2,188,029 clinical encounters by 285,305 patients, totaling 7,245,694 unique measurements, or observations. Eligible subjects were ≥18 years of age with information available on relevant behavioral and clinical factors needed to measure CVH. We used the first recorded measure of a factor of CVH of each patient in each year for the analyses, but multiple CVH factors measured for a patient may have been recorded at different outpatient visits. All participating health care systems provided consent that allowed for de-identified data to be used for research purposes. The Ohio State University IRB approved the study protocol.

We defined CVH using AHA criteria, with modifications for the measures of total cholesterol, fasting glucose, and cigarette smoking status (Appendix Table 1). Five measures were extracted from EHRs and were assigned a score to categorize them as ideal, intermediate, or poor health: 1) systolic and diastolic readings for blood pressure (BP), 2) body mass index (BMI) for adiposity, 3) plasma concentration of low-density lipoprotein (LDL) for cholesterol, 4) glycosylated hemoglobin (HbA1c) for diabetes mellitus (DM), and 5) cigarette smoking status. Thresholds for the ideal category of BP, cholesterol, and DM were strictly based on clinical values and reflected that the patient achieved these values without the use of medication. Patients were categorized into poor CVH for BP, cholesterol, and DM if the clinical value met that of the poor category regardless of whether the patient was pharmacologically treated, and into intermediate CVH if either 1) the clinical value met that of the intermediate category without pharmacological treatment or 2) the patient met the ideal category cut points but was pharmacologically treated. Medication names, and order and discontinuation dates, accounted for type and year of medication use. We considered patients to be pharmacologically treated if the years for the order and discontinuation dates captured the year of analysis. Relevant drugs for this analysis were those indicated by the AHA to manage hypertension and hypercholesterolemia, and those indicated by the ADA to manage DM [17,18,19]. Patients not prescribed any of these drugs were considered to not be medically treated.

**Table 1:** Demographic characteristics of patients and mean values of continuous CVH measures overall and by year of analysis for all eligible patients.

	2012	2013	2014	2015	Overall^1^

**Total number of patients, n**	73,618	110,227	116,619	117,997	203,488

**Sex**					

**Female, n (%)**	43,560 (59.2)	63,650 (57.8)	67,524 (57.9)	68,389 (58.0)	117,624 (57.8)
**Male, n (%)**	30,036 (40.8)	46,545 (42.2)	49,047 (42.1)	49,535 (42.0)	85,744 (42.2)
**Missing, n**	22	32	48	73	120
**Race**					

**White, n (%)**	36,545 (84.3)	60,222 (80.3)	64,982 (80.9)	63,109 (80.4)	103,212 (75.7)
**Non-white, n (%)**	6,804 (15.7)	14,825 (19.8)	15,314 (19.1)	15.437 (19.6)	33,059 (24.3)
**Missing, n**	30,269	35,180	36,323	39,451	67,217
**Age, years, mean (SD^2^)**	49.68 (18.2)	51.32 (18.5)	51.20 (18.4)	50.86 (18.3)	49.43 (18.4)^3^

**BMI, kg/m^2^, mean (SD)**	30.7 (7.6)	30.2 (7.1)	30.3 (7.1)	30.4 (7.1)	N/A

**BP**					

**Systolic, mm Hg, mean (SD)**	125.5 (17.8)	126.1 (18.4)	126.4 (18.5)	126.1 (18.5)	N/A
**Diastolic, mm Hg, mean (SD)**	77.1 (11.4)	76.2 (11.7)	76.5 (11.5)	76.3 (11.4)	N/A
**LDL, mg/dL, mean (SD)**	103.1 (33.8)	104.1 (33.3)	105.7 (34.1)	107.4 (34.4)	N/A

**HbA1c, percent, mean (SD)**	6.9 (1.7)	7.0 (1.8)	7.1 (1.9)	7.0 (1.9)	N/A

^1^ Each unique eligible patient from 2012 to 2015; counting each person only once, regardless of how many measures he/she may have contributed each year or over the range of the years of analysis. ^2^ SD = standard deviation. ^3^ Age in 2015.

Of the 7,245,694 observations, 1,297,678 recorded BMI, 2,001,628 recorded BP, 342,188 recorded LDL, 212,881 recorded HbA1c, and 3,391,319 recorded smoking status. We used up to seven different exclusion criteria to arrive at the final total of observations for analysis of each CVH component (Appendix Table 2). Observations were excluded due to repeats within the same year, falling outside of the analysis timeframe, missing values, no birthdate to determine age, implausible values, age <18 years old, and lack of medication information. Our final sample for analysis of CVH measures comprised 203,488 patients with 677,733 clinical visits. We used a total of 1,224,317 (235,671 BMI, 348,933 BP, 177,789 LDL, 80,549 HbA1c, and 381,375 cigarette smoking) measures for analyses.

### Statistical Analyses

First, we reported on the prevalence of patients in each CVH category for each metric in each year of observation. The Mantel-Haenszel chi-square test was performed to determine if prevalences were associated with time. We then conducted a closed cohort analysis in order to follow the same patients over time, and to compare trends in CVH over time among sex and race. We recoded race as a binary variable (white vs. non-white) and categorized sex as female or male. We first imputed scores forward for one year only for patients with missing scores. As an example, if a score were missing in 2015, we carried forward the score in 2014. If data was missing for a score in 2012, we then imputed backward the value of that score in 2013. In our sample of 203,488 patients, we excluded 199,094 observations with one or more score missing, and a further 2,504 observations that were missing race. The final closed cohort consisted of 1,890 patients with complete CVH and demographic data. We additionally conducted a sensitivity analysis to determine whether those included in the cohort differed from those who were excluded on key demographic variables. All analyses were performed using SAS 9.4 (SAS Institute, Inc.). Statistical significance was set at α = 0.05.

## Results

**Figure 1:**
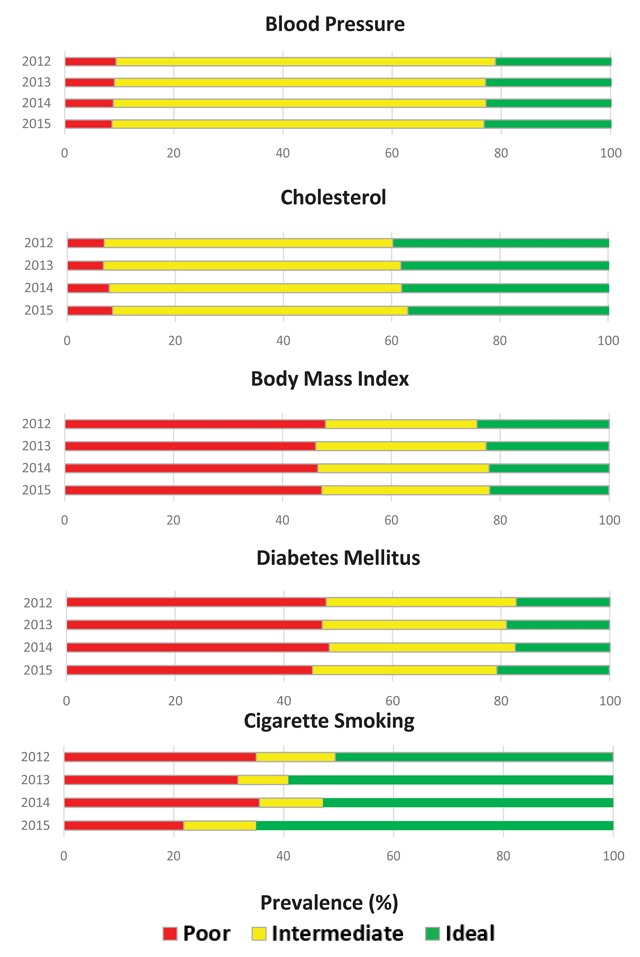
Prevalence of ideal, intermediate, and poor cardiovascular health for blood pressure, cholesterol, body mass index, diabetes mellitus, and cigarette smoking from 2012 to 2015 among United States adults ≥18 years of age in TGA (n = 203,488).

Patients included in the analysis (n = 203,488) were predominantly female (57.8 percent) and white (75.7 percent) with an average age of 49 years (standard deviation, SD = 18.4) (Table 1). The proportion of female and white patients, and their average age, remained relatively constant over time despite the gradual increase in sample size. We first calculated the annual prevalence (2012–2015) of CVH according to ideal, intermediate, and poor categories for each metric. There was significant overall changes in prevalence of categories for each metric over time (p < 0.0001). Ideal CVH improved for cigarette smoking (50.6 percent to 65 percent) and modestly improved for BP (21.1 percent to 23.2 percent) and DM (17.3 percent to 20.7 percent) from 2012 to 2015 (Figure 1). Slight decreases in ideal CVH were seen for cholesterol (39.8 percent to 37 percent) and BMI (24.3 percent to 21.9 percent). Cigarette smoking had the greatest proportion of patients with ideal CVH in all four years. Meanwhile, the majority of patients had poor health for BMI and DM and intermediate health for BP and cholesterol in all years of analysis.

Mean values of CVH measures for BMI, BP, cholesterol, and DM remained relatively stable over time (data not shown). Our most recent year of analysis indicates that patients seen in these clinics were, on average, obese (mean BMI, SD = 30.4 kg/m^2^, 7.1), pre-hypertensive (mean systolic BP, SD = 126.1 mm Hg, 18.5; mean diastolic BP, SD = 76.3 mm Hg, 11.4), pre-hypercholesterolemic (mean LDL, SD = 107.4 mg/dL, 34.4), and met the clinical cut point for diabetes (mean HbA1c, SD = 7.0 percent, 1.9). We observed that average values corresponded to the most prevalent category of CVH in 2015 for BMI (poor), BP (intermediate), cholesterol (intermediate), and DM (poor).

**Figure 2:**
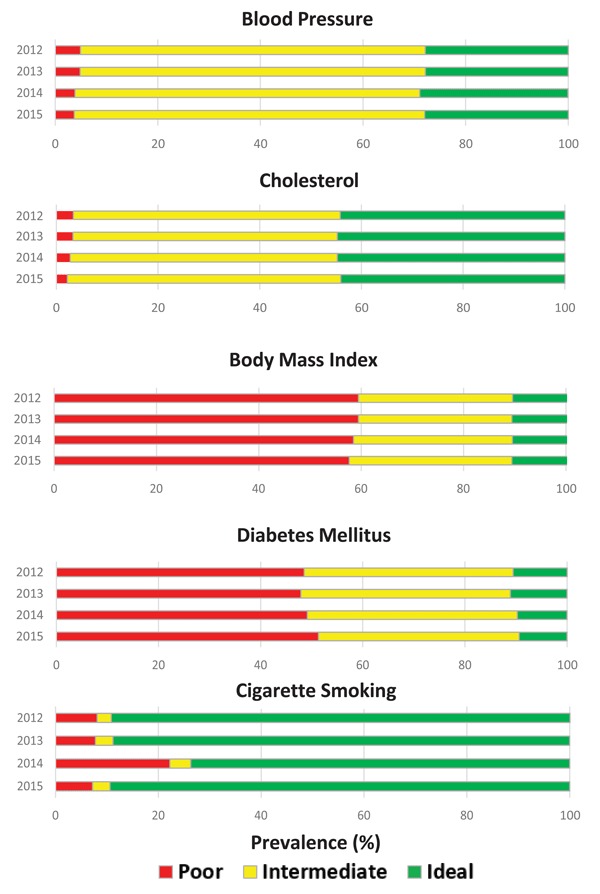
Prevalence of ideal, intermediate, and poor cardiovascular health for blood pressure, cholesterol, body mass index, diabetes mellitus, and cigarette smoking from 2012 to 2015 among the closed cohort in TGA (n = 1890).

The sample for the closed cohort analysis was 42.7 percent (n = 807) female and 81.6 percent (n = 1543) white with a mean age of 63.0 years (SD = 12.7) at baseline. There was a significantly greater proportion of females (p < 0.0001) and whites (p < 0.0001) who were eligible for the closed cohort compared to those who were not seen as a patient for four consecutive years. Patients in the cohort were also significantly older at baseline (p < 0.0001). In this patient subset, the only change we saw over time in prevalence of ideal CVH for a measure after four years was a decrease for DM (10.6 percent to 9.4 percent) (Figure 2). Meanwhile, the prevalence of poor CVH decreased over time for BP (4.9 percent to 3.7 percent), cholesterol (3.3 percent to 2.2 percent), and BMI (59.5 percent to 57.7 percent).

**Figure 3:**
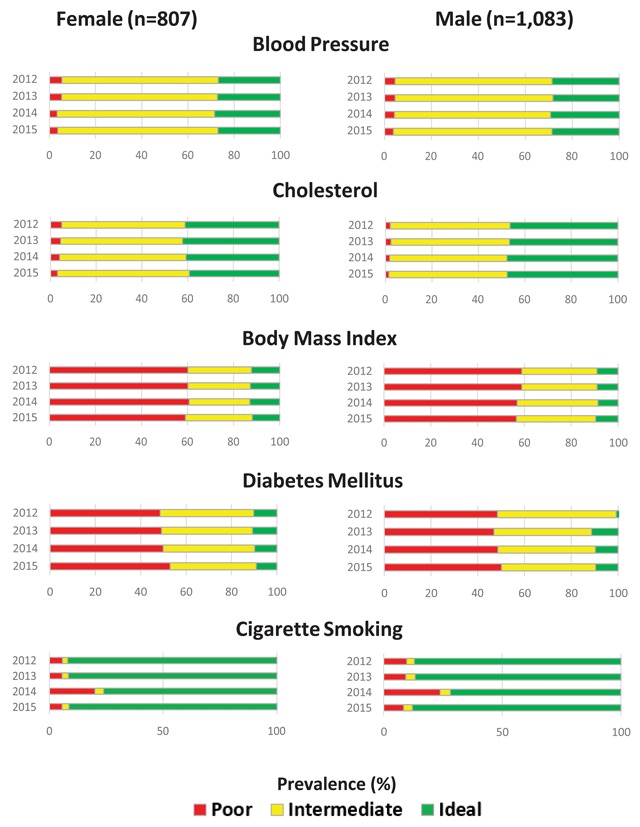
Prevalence of ideal, intermediate, and poor cardiovascular health for blood pressure, cholesterol, body mass index, diabetes mellitus, and cigarette smoking from 2012 to 2015 in the closed cohort, stratified by sex.

**Figure 4:**
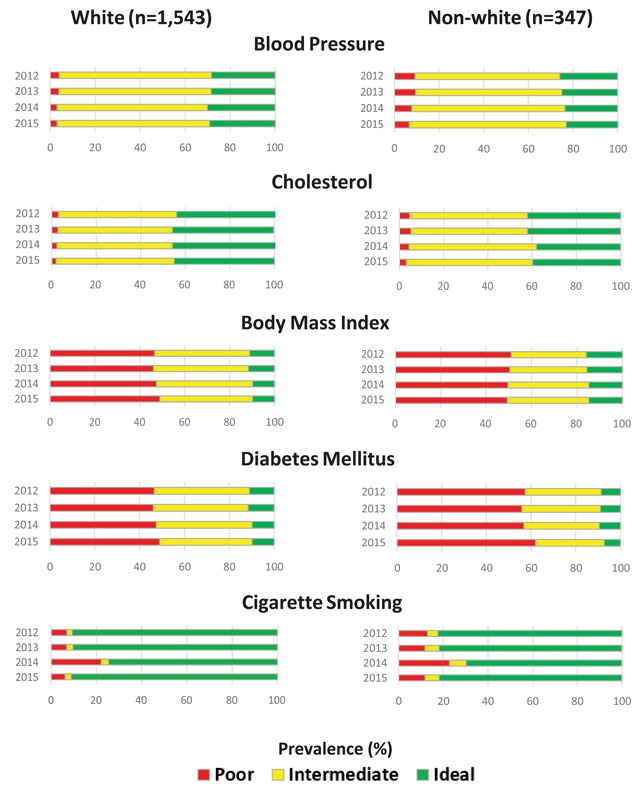
Prevalence of ideal, intermediate, and poor cardiovascular health for blood pressure, cholesterol, body mass index, diabetes mellitus, and cigarette smoking from 2012 to 2015 in the closed cohort, stratified by race.

Stratified analyses indicated that prevalence of ideal CVH for males was higher for BP, cholesterol, and DM compared to females at baseline and at follow-up (Figure 3). Ideal CVH for females did not improve over time for any CVH metric, but cholesterol (46.5 percent to 47.7 percent) did improve over the same time period for males. A greater proportion of white patients had ideal CVH at baseline and follow-up for BP, cholesterol, DM, and cigarette smoking compared to non- whites (Figure 4). Ideal CVH for BP (25.9 percent to 23.1 percent), cholesterol (42.1 percent to 39.8 percent), BMI (15.9 percent to 14.7 percent), and DM (8.65 percent to 7.2 percent) decreased over time for non-whites; only DM decreased over time for whites (11.0 percent to 9.9 percent). White patients saw modest increases in ideal CVH over time for BP (28.3 percent to 29.1 percent), cholesterol (44.7 percent to 45.0 percent), and BMI (9.2 percent to 9.7 percent).

## Discussion

The ability to pool EHR data from numerous health systems across the US is a novel feature of TGA. This is the first time the CVH of Americans has been described over time using pooled EHR data in TGA repository. CVH metrics that showed improvement over the 4-year period included BP (21.1 percent to 23.2 percent ideal), DM (17.3 percent to 20.7 percent ideal), and cigarette smoking (50.6 percent to 65.9 percent ideal), while cholesterol (39.8 percent to 37.0 percent ideal) and BMI (24.3 percent to 21.9 percent ideal) worsened. Furthermore, men were consistently healthier than women for BP and cholesterol and whites had a noticeably higher prevalence of ideal CVH for BP, cholesterol, DM, and cigarette smoking than non-whites. Progress towards AHA’s 2020 Impact Goal is not demonstrated in the outpatient setting, as strides made towards ideal CVH according to some metrics are offset by exacerbations of others.

Public health researchers monitor trends in the proportion of Americans within each health category to evaluate progress towards AHA’s 2020 Impact Goals. The most recent assessment of current CVH and health projections was based on 35,059 participants aged ≥20 years and free of CVD from the National Health and Nutrition Examination Survey (NHANES) III and the 1999-2008 continuous 2 year cycles [15]. Men and women saw improvements in ideal CVH for cigarette smoking only, and worsening for BMI and DM over time. Our most recent prevalence estimates of ideal CVH for BMI (9.6 percent), BP (28.7 percent), and DM (9.6 percent) over time among men were considerably smaller than what was projected with NHANES data for 2020 (16.8 percent, 40.1 percent, and 23.3 percent, respectively). Among women, our estimates were also lower for ideal BMI (11.9 percent), BP (27.0 percent), DM (9.05 percent), and cholesterol (39.3 percent) compared to that of NHANES (27.9 percent, 48.9 percent, 47.4 percent, and 45.5 percent, respectively). Differences may be explained by attributes in each population, including health status and medication. Patients from the outpatient setting are likely to be more ill than those from the general population because those with chronic conditions tend to seek medical care compared to those who are generally healthy [20]. Additionally, the prevalence of prescription drug use remained relatively stable over time in the outpatient setting, but increased over time among national survey samples [21].

Limitations to the use of EHR data from the TGA repository for this purpose should be mentioned. Due to staggered entry of health care systems into TGA and the obligatory nature of submitting data, not all provided data on all metrics in the years of analysis. Health care systems were also free to utilize different EHR software, which led to inconsistent collection and recording of cigarette smoking status. Two systems did not record history of former cigarette smoking and we were limited to report only on current and nonsmoking behaviors of their patients. EHR systems that permitted free text to document cigarette smoking were scrutinized, and subsequently more laborious to recode compared to those with drop-down menus of fixed options for responses. A consistent method of recording smoking in the EHR can resolve issues that arise when capturing CVH of patients in public health research that uses pooled EHR data from multiple health care systems. Regardless, data harmonization is essential when utilizing EHR data such as TGA, which combined metrics measured non-uniformly across different health care systems.

Metrics needed to ascertain CVH include clinical measures and behavioral factors, some of which can be found in the EHR. Notably, our analysis lacked information on diet and physical activity (the other two components of CVH), since those data are not routinely captured in the EHR. Another limitation of our analysis is that total cholesterol and fasting glucose was not available for patients. Instead, we used the available measures of LDL and HbA1c, metrics more commonly collected and used in the clinical setting, and used clinically-relevant cut points to assign patients to the poor, intermediate, or ideal category for cholesterol and DM metrics [22,23]. While we benefited from using CVH measures routinely captured at outpatient visits, we were simultaneously challenged by variable numbers of measures within a patient in the same year. Additionally, medication adherence remains an issue despite having access to order and discontinuation dates of prescriptions. The data set also contained a substantial amount of observations missing data for CVH metrics, possibly attributed to the difficult nature of smoothly harmonizing data, or because clinicians did not order a test. These aspects were encompassed by our rigorous exclusion criteria, which left us unable to incorporate a substantial amount of observations into our analysis. This may limit the representativeness of the patients to the US adult population, and may have led us to incorrectly estimate their CVH. Since this is secondary data, we had no control over the missingness and are unable to determine exactly what caused its abundance. Also due to the nature of secondary analyses, we were unable to distinguish outpatient visit types, and are concerned with the possibility that patient data recorded at surgical or emergency department outpatient visits were in the TGA repository and may have biased our estimates.

Studies using EHR data must be thoughtfully designed. A strength of our study is that we used an evidence-based CVH metric, along with patient data to characterize the CVH of a large patient population. Challenges in using EHR data for research purposes may include sample representativeness [24], intermittent data collection intervals, and missing data. Using our methodology, TGA data can be re-evaluated prospectively to assess progress in the outpatient setting toward the 2020 Impact Goal and to quantify adherence to relevant clinical guidelines. However, the degree of missingness, our sensitivity analysis, and non-probability selection of health care systems indicates that imputation and application of additional methods to weight the data set may be needed to achieve representativeness of the target population. Future analyses should also perform equivalence testing to compare our CVH estimates with those obtained from NHANES or other nationally representative samples.

## Conclusion

Our analysis supports how electronic documentation of markers of CVH in the outpatient setting can transform how we measure population health in observational studies. We established the utility of EHR data to evaluate risk factor burden and identify vulnerable subpopulations and CVH disparities. This research confirms that the majority of Americans still need to improve their CVH, and patients and providers can work together to accomplish better health outcomes. The proportion of Americans in the outpatient setting with ideal CVH for all measures is less than desired, but this is especially apparent for BP, BMI, cholesterol, and DM. Strategies that augment adherence to guideline-based care are warranted to achieve a positive impact on this threat to public health.

## Additional File

The additional file for this article can be found as follows:

10.5334/egems.268.s1 Appendix 1. EHR-derived CVH Metrics and Exclusion Criteria.

## References

[1] Centers for Disease Control and Prevention: Division for Heart Disease and Stroke Prevention. Heart Disease Facts. 2016 [cited 2017 October 31]. Available from: http://www.cdc.gov/dhdsp/data_statistics/fact_sheets/fs_heart_disease.htm.

[2] Taksler, GB and Rothberg, MB. Assessing Years of Life Lost Versus Number of Deaths in the United States, 1995–2015. American Journal of Public Health. 2017; 107(10): 1653–9. DOI: 10.2105/AJPH.2017.303986 28817329 PMC5607680

[3] Loprinzi, PD, Branscum, A, Hanks, J and Smit, E. Healthy Lifestyle Characteristics and Their Joint Association With Cardiovascular Disease Biomarkers in US Adults. Mayo Clinic Proceedings. 91(4): 432–42. DOI: 10.1016/j.mayocp.2016.01.009 26906650

[4] Casey, JA, Schwartz, BS, Stewart, WF and Adler, NE. Using Electronic Health Records for Population Health Research: A Review of Methods and Applications. Annual Review of Public Health. 2016; 37(1): 61–81. DOI: 10.1146/annurev-publhealth-032315-021353 PMC6724703 26667605

[5] Foraker, RE, Shoben, AB, Kelley, MM, Lai, AM, Lopetegui, MA, Jackson, RD, et al. Electronic health record-based assessment of cardiovascular health: The stroke prevention in healthcare delivery environments (SPHERE) study. Preventive Medicine Reports. 2016; 4: 303–8. DOI: 10.1016/j.pmedr.2016.07.006 27486559 PMC4959947

[6] Bilal, U, Díez, J, Alfayate, S, Gullón, P, del Cura, I, Escobar, F, et al. Population cardiovascular health and urban environments: the Heart Healthy Hoods exploratory study in Madrid, Spain. BMC Medical Research Methodology. 2016; 16(1): 104 DOI: 10.1186/s12874-016-0213-4 27549991 PMC4994419

[7] Al-Kindi, SG and Oliveira, GH. Prevalence of Preexisting Cardiovascular Disease in Patients With Different Types of Cancer: The Unmet Need for Onco-Cardiology. Mayo Clinic Proceedings. 2016; 91(1): 81–3. DOI: 10.1016/j.mayocp.2015.09.009 26602599

[8] Incidence and prevalence of select cardiovascular risk factors and conditions, active component, U.S. Armed Forces, 2003–2012. 2013 [cited 2017 October 31]. Available from: https://health.mil/Military-Health-Topics/Health-Readiness/Armed-Forces-Health-Surveillance-Branch/Reports-and-Publications/Medical-Surveillance-Monthly-Report/View-Past-Reports. 24428539

[9] Asaria, M, Walker, S, Palmer, S, Gale, CP, Shah, AD, Abrams, KR, et al. Using electronic health records to predict costs and outcomes in stable coronary artery disease. Heart. 2016; 102(10): 755–62. DOI: 10.1136/heartjnl-2015-308850 26864674 PMC4849559

[10] Green, BB, Anderson, ML, Cook, AJ, Catz, S, Fishman, PA, McClure, JB, et al. Using Body Mass Index Data in the Electronic Health Record to Calculate Cardiovascular Risk. American Journal of Preventive Medicine. 2012; 42(4): 342–7. DOI: 10.1016/j.amepre.2011.12.009 22424246 PMC3308122

[11] Reeves, MJ and Rafferty, AP. Healthy lifestyle characteristics among adults in the United States, 2000 Archives of Internal Medicine. 2005; 165(8): 854–7. DOI: 10.1001/archinte.165.8.854 15851634

[12] Sidebottom, AC, Johnson, PJ, Van Wormer, JJ, Sillah, A, Winden, TJ and Boucher, JL. Exploring Electronic Health Records as a Population Health Surveillance Tool of Cardiovascular Disease Risk Factors. Population Health Management. 2015; 18(2): 79–85. DOI: 10.1089/pop.2014.0058 25290223

[13] Bufalino, V, Bauman, MA, Shubrook, JH, Balch, AJ, Boone, C, Vennum, K, et al. Evolution of “The Guideline Advantage”: Lessons learned from the front lines of outpatient performance measurement. CA: A Cancer Journal for Clinicians. 2014; 64(3): 157–63. DOI: 10.3322/caac.21233 24788583

[14] Lloyd-Jones, DM, Hong, Y, Labarthe, D, Mozaffarian, D, Appel, LJ, Van Horn, L, et al. Defining and Setting National Goals for Cardiovascular Health Promotion and Disease Reduction. The American Heart Association’s Strategic Impact Goal Through 2020 and Beyond. 2010; 121(4): 586–613. DOI: 10.1161/CIRCULATIONAHA.109.192703 20089546

[15] Huffman, MD, Capewell, S, Ning, H, Shay, CM, Ford, ES and Lloyd-Jones, DM. Cardiovascular Health Behavior and Health Factor Changes (1988–2008) and Projections to 2020: Results From the National Health and Nutrition Examination Surveys. Circulation. 2012; 125(21): 2595–602. DOI: 10.1161/CIRCULATIONAHA.111.070722 22547667 PMC3914399

[16] The Guideline Advantage. About the Guideline Advantage. 2013 [cited 2017 October 31]. Available from: http://www.guidelineadvantage.org/TGA/About/About_UCM_428634_Article.jsp.

[17] American Heart Association. High Blood Pressure. Types of Blood Pressure Medications. 2016 [cited 2017 January 30]. Available from: http://www.heart.org/HEARTORG/Conditions/HighBloodPressure/PreventionTreatmentofHighBloodPressure/Types-of-Blood-Pressure-Medications_UCM_303247_Article.jsp#.WZH6ek2FOpo.

[18] American Heart Association. Cholesterol. Cholesterol Medication. 2016 [cited 2017 January 30]. Available from: http://www.heart.org/HEARTORG/Conditions/Cholesterol/PreventionTreatmentofHighCholesterol/Cholesterol-Medications_UCM_305632_Article.jsp#.WZH6GU2FOpo.

[19] American Diabetes Association. Living with Diabetes. Treatment and Care: Medication. 2016 [cited 2017 January 30]. Available from: http://www.diabetes.org/living-with-diabetes/treatment-and-care/medication/?referrer=https://www.google.com/.

[20] Rusanov, A, Weiskopf, NG, Wang, S and Weng, C. Hidden in plain sight: bias towards sick patients when sampling patients with sufficient electronic health record data for research. BMC Medical Informatics and Decision Making. 2014; 14: 51 DOI: 10.1186/1472-6947-14-51 24916006 PMC4062889

[21] Kantor, ED, Rehm, CD, Haas, JS, Chan, AT and Giovannucci, EL. Trends in prescription drug use among adults in the united states from 1999–2012. JAMA. 2015; 314(17): 1818–30. DOI: 10.1001/jama.2015.13766 26529160 PMC4752169

[22] Third Report of the National Cholesterol Education Program (NCEP) Expert Panel on Detection, Evaluation, and Treatment of High Blood Cholesterol in Adults (Adult Treatment Panel III) Final Report. Circulation. 2002; 106(25): 3143 DOI: 10.1161/circ.106.25.3143 12485966

[23] American Diabetes Association. Diagnosis and Classification of Diabetes Mellitus. Diabetes Care. 2010; 33(Suppl 1): S62–S9. DOI: 10.2337/dc10-S062 20042775 PMC2797383

[24] Bower, JK, Bollinger, CE, Foraker, RE, Hood, DB, Shoben, AB and Lai, AM. Active Use of Electronic Health Records (EHRs) and Personal Health Records (PHRs) for Epidemiologic Research: Sample Representativeness and Nonresponse Bias in a Study of Women During Pregnancy. eGEMs. 2017; 5(1): 1263 DOI: 10.13063/2327-9214.1263s 28303255 PMC5340503

