# Supplementary material for: Cardiovascular Health Trends in Electronic Health Record Data (2012–2015): A Cross-Sectional Analysis of The Guideline Advantage™
Source: EGEMS (Wash DC). 2019 Jul 18;7(1):30. doi: 10.5334/egems.268 (PMC6646939; doi:10.5334/egems.268)
Supplement: Appendix 1. — EHR-derived CVH Metrics and Exclusion Criteria. [file egems-7-1-268-s1.pdf]

Appendix

Patients were considered treated for elevated BP if one or more of their prescribed drugs were of the following classes: diuretics, beta-blockers, angiotensin-converting enzyme inhibitors, angiotensin II receptor blockers, calcium channel blockers, alpha blockers, alpha-2 receptor agonists, combined alpha and beta-blockers, central agonists, peripheral adrenergic inhibitors, or vasodilators. If a drug name was classified as a statin, selective cholesterol adsorption inhibitors, resins, or lipid-lowering therapy drug, we considered the patient to be treated for high cholesterol. Patients treated for diabetes mellitus were those prescribed drugs from the insulin, sulfonylureas, iguanids, meglitinides, thiazolidinediones, DPP-4 inhibitors, SGLT2 inhibitors, alpha-glucosidase inhibitors, or bile acid sequestrants classes of medications.

For observations recording BMI, we excluded 113,718 because they did not fall within our timeframe of analysis, 23,982 due to implausible values, 91,725 due to lack of age information, 158,932 due to missing values, 669,111 due to repeat measurement within that year, and 4,539 because the patient was <18 years old. For observations recording BP, we excluded 72,983 because they did not fall within our timeframe of analysis, 49,751 due to implausible values, 177,238 due to lack of age information, 78,068 due to missing values, 1,220,935 due to repeat measurement within that year, and 8291 because the patient was <18 years old, and 45,429 due to lack of medication data. For observations recording LDL, we excluded 38,752 because they did not fall within our timeframe of analysis, 17,945 due to implausible values, 2,270 due to lack of age information, 5,009 due to missing values, 92,351 due to repeat measurement within that year, 399 because the patient was <18 years old, and 7,673 due to lack of medication data. For observations recording HbA1c, we excluded 19,738 because they did not fall within our timeframe of analysis, 17,555 due to implausible values, 58,310 due to lack of age information, 3,293 due to missing values, 88,056 due to repeat measurement within that year, 275 because the patient was <18 years old, and 3,415 due to lack of medication data. For observations recording smoking status, we excluded 150,185 because they did not fall within our timeframe of analysis,

2,145,768 due to missing data, 63,557 due to lack of age information, 643,561 due to repeat measurement within that year, and 6,873 because the patient was <18 years old.

Appendix Table 1. Definitions of poor, intermediate, and ideal categories for each CVH measure

	**Poor**	**Intermediate**	**Ideal**
**Body mass index (BMI)**	≥30 kg/m^2	25 to <30 kg/m^2	<25 kg/m^2
**Blood pressure (BP)**	Systolic ≥140 mm Hg or diastolic ≥90 mm Hg	Systolic 120-139 mm Hg, diastolic 80-89 mm Hg, or treated to goal	Systolic <120 mm Hg or diastolic <80 mm Hg
**Low-density lipoprotein (LDL)**	≥160 mg/dL	100-159 mg/dL or treated to goal	<100 mg/dL
**Glycosylated hemoglobin (HbA1c)**	≥6.5%	6.0-6.4%	<6.0%
**Cigarette smoking status**	Current	Former	Never

Appendix Table 2. Quantity of observations excluded at each data management step.

	Repeat measure	Outside of year range	Missing values	No birthdate for age	Implausible values[1]	<18 years old	Medication
**Body mass index (BMI)**	669,111	113,718	158,932	91,725	23,982	4,539	N/A[2]
**Blood pressure (BP)**	1,220,935	72,983	78,068	177,238	49,751	8291	45,429
**Low-density lipoprotein (LDL)**	92,351	38,752	5,009	2,270	17,945	399	7,673
**Glycosylated hemoglobin (HbA1c)**	88,056	19,738	3,293	58,310	17,555	275	3415
**Cigarette smoking**	643,561	150,185	2,145,768	63,557	N/A	6,873	N/A

[1]Implausible values for body mass index were those <11.55 kg/m^2 or >55.00 kg/m^2; for blood pressure were systolic <20 mm Hg or >260 mm Hg, diastolic <20 mm Hg or >200 mm Hg, or pulse pressure <20 mm Hg; for low-density lipoprotein were those <20 mg/dL or >220 mg/dL; and for glycosylated hemoglobin were those <2% or >14%.

[2]N/A= not available
